# Implementation and Evaluation of a Virtual Transitional Care Intervention Using Automated Text Messaging and Virtual Visits After Emergency Department Discharges: Retrospective Cohort Study

**DOI:** 10.2196/77973

**Published:** 2025-10-29

**Authors:** Grace Lee, Courtenay Bruce, Tariq Nisar, Brendan Holderread, Sarah Pletcher, Ngoc Anh Nguyen

**Affiliations:** 1 Center for Connected Care, Innovation & Implementation - Research, Houston Methodist Hospital, 6445 Main Street, Houston, TX, 77030, United States, 1 7137931851; 2 Houston Methodist Academic Institute, Houston Methodist Hospital, Houston, TX, United States; 3 System Quality & Patient Safety, Houston Methodist Hospital, Houston, TX, United States; 4 Department of Surgery, Houston Methodist Hospital, Houston, TX, United States; 5 Department of Medicine, Houston Methodist Hospital, Houston, TX, United States

**Keywords:** transitional care intervention, SMS text messaging, virtual care, telemedicine, emergency department, hospital readmission, postdischarge follow-up

## Abstract

**Background:**

Emergency department (ED) overcrowding and avoidable revisits challenge health systems, with approximately 20% of patients returning within 30 days. ED-based transitional care interventions, including automated SMS text messaging, offer scalable, cost-effective means to improve follow-up, though evidence remains limited.

**Objective:**

This study evaluated a transitional care intervention combining SMS text messaging and virtual transitional care visits to reduce ED revisits and improve outpatient follow-up.

**Methods:**

This retrospective observational cohort study included patients discharged from 4 EDs within a single US health system between September 2023 and September 2024. Patients were categorized into two groups based on intervention engagements: (1) completed (requested, scheduled, and completed a visit) and (2) noncompleted (requested, scheduled, and did not complete). The primary outcome was spontaneous, unplanned ED revisits within 90 days; secondary outcomes included outpatient follow-up and time to first outpatient evaluation. Between-group differences were assessed using descriptive statistics and multivariable regression models (with *P*<.05 considered statistically significant).

**Results:**

Of 68,115 discharged patients, 42.72% (29,100/68,115) received an automated SMS text messaging for the virtual transitional care program, and 2.93% (853/29,100) accessed the scheduling link. Of these, 56.5% (482/853) requested a visit, 49.8% (240/482) scheduled, and 70% (168/240) completed the visit (completed group). Among 72 noncompleted patients, 57% (n=41) did not show, 32% (n=23) canceled, and 11% (n=8) scheduled 2 appointments but completed neither. Nearly half (35/72, 49%) of the noncompleted group had a subsequent ambulatory follow-up. Demographics, comorbidities, and acuity were similar. The noncompleted group was nearly twice as likely to return to the ED within 90 days (21/72, 29% vs 28/150 18.7%; *χ*^2^_1_=4.20, *P*=.04; odds ratio 2.11, 95% CI 1.02‐4.33), while the completed group was more likely to complete outpatient follow-up (35/72, 49% vs 51/168, 30.4%; *χ*^2^_1_=6.60, *P*=.01; odds ratio 2.15, 95% CI 1.03‐4.77). Time to first outpatient visit did not differ significantly between groups (mean 15.7, SD 19.0 d vs mean 19.8, SD 20.7 d; Δβ=–1.93, 95% CI –10.09 to 6.42; *P*=.65).

**Conclusions:**

A combined SMS text messaging and virtual transitional care program lowered 90-day ED revisits and increased outpatient follow-up, but engagement was low (2.9%). Future work should focus on optimizing care delivery and developing strategies to expand reach across the broader ED discharge population.

## Introduction

Emergency department (ED) overcrowding and avoidable return visits represent significant challenges for health care systems, with approximately 20% of patients returning to the ED within 30 days of discharge [1]. ED return visits can signal gaps in postdischarge care coordination, unresolved clinical concerns, or inadequate follow-up, all of which contribute to worse patient outcomes and increased strain on health care resources. Notably, about 14% of ED return visits result in hospital admission, underscoring the role of the ED as a critical entry point for both initial and subsequent acute care use [2]. These findings suggest that timely post-ED follow-up could help prevent return visits and downstream hospitalizations. This dual impact—reducing ED overcrowding while preventing readmissions—makes ED-discharged patients a strategic target for transitional care interventions (TCIs).

A wide range of TCIs have been implemented to reduce avoidable acute care reuse with varying outcomes. Timely face-to-face primary care follow-ups have been shown to reduce readmissions, but only 30% to 49% of patients secure an appointment within 14 to 28 days after discharge [3,4]. This gap is often even wider among patients discharged from the ED, who may lack a primary care provider, health insurance, or familiarity with outpatient care navigation, factors that further complicate successful follow-up. Telephone outreach is an area of potential promise that has previously demonstrated mixed results. A retrospective cohort study analyzing all patients discharged from 15 Californian hospitals over 2 years found that telephone follow-up had a marginal impact on 30-day readmissions in high-risk medicine patients and no effect in low-risk patients [5]. In contrast, Constantino et al [6] demonstrated that telephone intervention reduced ED revisits and 30-day hospital readmissions in Medicare Advantage beneficiaries enrolled in a large national health care company. Other TCIs, such as postdischarge home visits, multidisciplinary transition care clinics, and pharmacist-led medication management, have been either ineffective overall, effective only in specific subgroups, or inconclusive due to limited enrollment [7-9].

These inconsistent outcomes, likely driven by the heterogeneity of patient populations across prior studies, combined with the labor-intensive and logistically complex nature of these interventions, raise concerns about their scalability and long-term sustainability. These challenges highlight the need for a TCI following acute care or ED visits that is effective; cost-efficient; and (1) broadly implementable across diverse patient populations while allowing flexibility for individual patient needs and preferences, (2) capable of providing timely follow-up without delays, and (3) scalable across different health care settings and clinical contexts. SMS text messaging and virtual transitional care visits have significant potential to address these gaps. SMS text messaging is cost-effective, widely accessible to all patients with mobile devices, and convenient due to its asynchronous nature [10]. SMS text messaging outreach further enables clinicians to target defined patient populations or permit text recipients to self-select into TCI. This flexibility facilitates the identification of patients most likely to benefit from additional postdischarge care navigation, thereby optimizing resource use while maintaining broad implementability.

In this study, we describe the conceptualization, development, implementation, and outcomes of a novel virtual TCI that integrates automated unidirectional SMS text messaging with virtual transitional care visits. While the visits were staffed by the system’s existing virtual urgent care (VUC) team, this was not a standard on-demand VUC encounter. Recognizing that the ED frequently represents the first step in the hospital readmission cycle, this intervention specifically targeted patients discharged directly from the ED through a virtual transitional care program designed to reconnect them with timely care, reinforce discharge instructions, and route unresolved concerns to appropriate next steps.

## Methods

### Study Design and Setting

This retrospective observational cohort study analyzed patients discharged from four EDs within our health system between September 2023 and September 2024. This study was reviewed and classified as a quality improvement initiative and “Not Human Research” by the health system’s institutional review board.

The study was conducted within a nonprofit health system in Houston, Texas, consisting of 1 academic quaternary hospital (1020 operating beds), 6 community hospitals (average of 277 beds each), and 1 long-term acute care facility (98 beds) [11]. The academic hospital also receives patients from 3 surrounding freestanding emergency care centers (ECCs).

### VUC Program Background

The system launched its VUC program in 2019 as part of an institutional innovation initiative to expand access to on-demand care through virtual platforms. For standard urgent care needs, patients can directly schedule VUC appointments online through the health system’s patient portal for new acute complaints. The VUC service operates 24/7/365 and is staffed by family nurse practitioners under the supervision of an emergency medicine physician. Patients can connect with providers via interactive, 2-way audio-video from their homes, offering convenient, on-demand care. In addition to treating the usual urgent care conditions, the program also facilitates referrals for diagnostics, establishing care with a primary care physician, and specialty care referrals. During the COVID-19 pandemic, VUC was also used to assess and refer patients to the health system’s monoclonal antibody infusion program. Establishing accessible referral pathways was critical to managing high patient volumes, and the VUC program served as a reliable source alongside freestanding ECCs and hospital-based EDs [12].

By April 28, 2019, the program had completed over 4500 VUC visits, with an average patient satisfaction score of 4.9 out of 5 [13]. Building on this early success, the VUC infrastructure and staffing model were adapted in 2023 to support a distinct use case: virtual transitional care visits for patients recently discharged from the ED. This adaptation was designed to leverage existing virtual care capabilities to address gaps in post-ED discharge care and represents the intervention evaluated in this study. Unlike standard VUC encounters, which address new symptoms, the virtual transitional care visits described in this study were initiated through our ED discharge workflows and specifically focused on postdischarge care coordination and assessment of ongoing concerns related to the initial ED visit.

### Participants

#### Eligibility Criteria

##### Inclusion Criteria

Eligibility required a documented phone number in the Epic electronic medical record (EMR) system (Epic Systems Corp) and aged ≥2 years. SMS text messaging was covered under the hospital-wide consent for postdischarge contact obtained at ED registration. Not all ED-discharged patients during the study period (September 2023 to September 2024) were assigned to receive automated SMS text messaging due to phased implementation and evolving eligibility criteria. During the pilot phase (September 2023 to February 2024), assignment to the virtual transitional care program was limited to patients diagnosed with chronic obstructive pulmonary disease, pneumonia, upper respiratory infection, or COVID-19. This respiratory-focused pilot approach served dual purposes: enabling targeted VUC nurse practitioner training in respiratory care management and chest imaging interpretation while establishing standardized virtual transitional care protocols for visit workflows, documentation, and care coordination before expanding to all ED discharge diagnoses. Beginning in March 2024, virtual transitional care pathway assignment expanded to include all discharged patients, regardless of diagnosis.

##### Exclusion Criteria

Patients who were directed to return to the ED by a VUC nurse practitioner were excluded from the outcome analysis. These cases represented provider-directed referrals for emergent evaluation, distinct from the spontaneous, patient-initiated ED revisits that this study sought to assess.

### Group Classification

Patients were categorized into two comparison groups based on their level of engagement with the virtual TCI:

1. Completed virtual transitional care visit group: patients who received an automated SMS text messaging, requested and scheduled a virtual transitional care visit through a patient service representative (PSR), and *completed* the visit. These patients are referred to as the “completed group” or “completed patients” throughout this study.
2. Noncompleted virtual transitional care visit group: Patients who received the SMS text messaging, requested and scheduled a virtual transitional care visit with a PSR, but *did not complete* the visit (due to a cancelation or no-show). These patients are referred to as the “noncompleted group” or “noncompleted patients” throughout this paper.

### Intervention: Virtual Transitional Care Program

The virtual transitional care program was developed with the following objectives: (1) direct patients with nonemergent concerns to alternate care options to reduce ED overcrowding and boarding times, (2) facilitate timely transitions to outpatient care through the existing VUC infrastructure, (3) improve continuity of care and prevent patients from becoming “lost to follow-up” after ED discharge, (4) enhance patient satisfaction through additional resources and referrals, and (5) provide timely clinical reassessment by trained clinicians.

#### Program Workflow and Technology Platform

**Figure 1.**
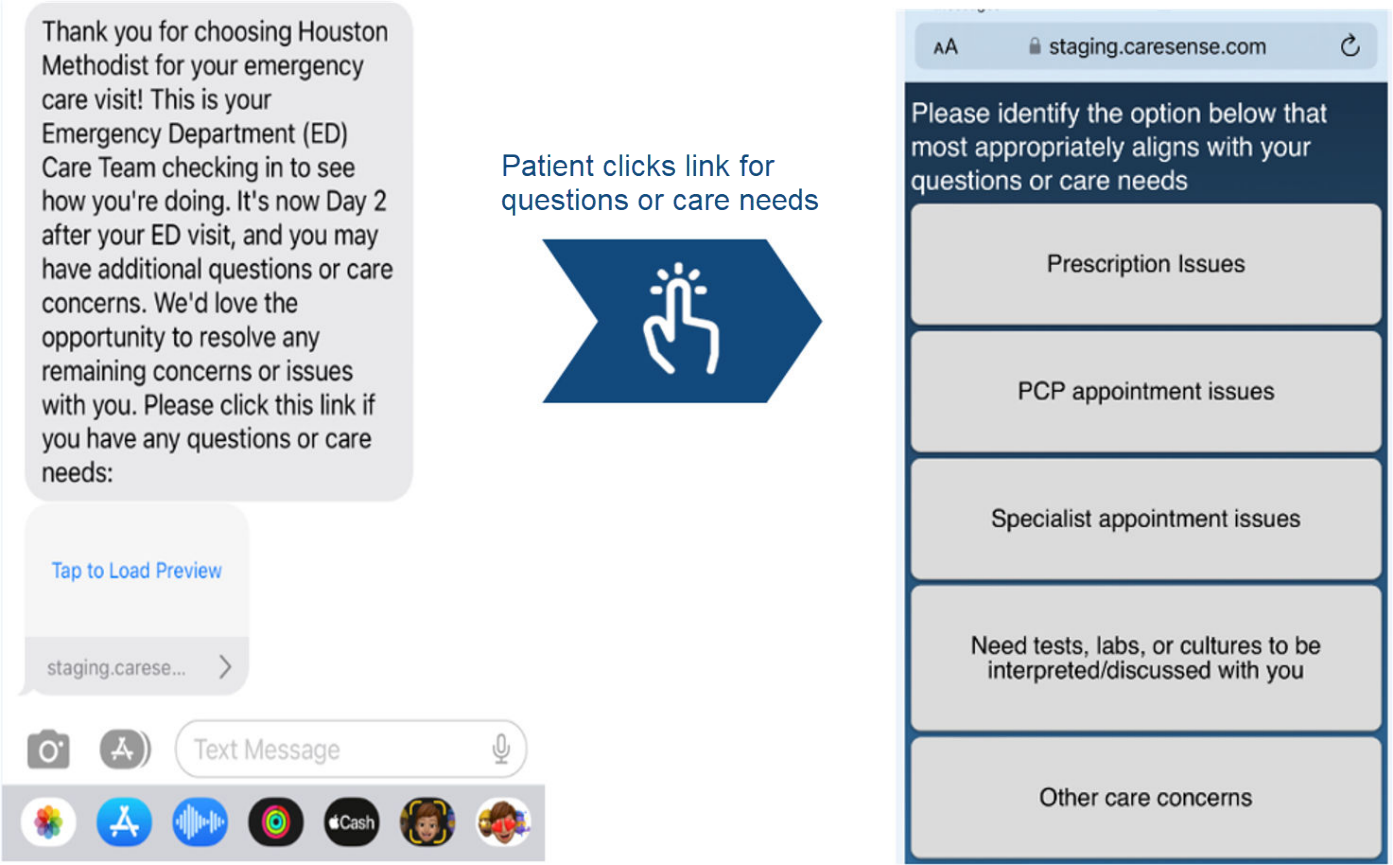
Day 2 postdischarge SMS text messaging and care needs survey interface platform. Clinical concerns route to virtual transitional care scheduling, while administrative concerns route to referral management. PCP: primary care physician.

The program leveraged *CareSense* (MedTrak, LLC), a third-party digital engagement platform, configured to align with institutional security and communication protocols. Within 48 hours of discharge, eligible patients receive an automated SMS text message with a link to a brief questionnaire (Figure 1).

Patient responses trigger different care pathways based on concern type. Clinical concerns (prescription issues, need for tests, laboratory results discussion, or “other”) route patients to PSRs who schedule virtual transitional care visits through Epic or MyChart infrastructure. Administrative concerns (appointment scheduling issues) redirect patients to the referral management center for outpatient appointment scheduling, bypassing the virtual transitional care pathway.

#### Virtual Transitional Care Visit Process

For patients routed to virtual transitional care, their medical record numbers are queued in the CareSense database for PSR review, who then contact patients by phone to confirm ongoing concerns and schedule virtual transitional care visits. Although visits are staffed by the health system’s VUC clinicians, these are structured follow-ups specifically designed for post-ED discharge care coordination rather than standard urgent care encounters addressing new acute symptoms. VUC nurse practitioners assess patients clinically to determine whether care plans require continuation, modification, or escalation to an in-person evaluation.

### Outcomes and Variables

#### Primary Outcome

The primary outcome was the occurrence of spontaneous ED revisits within 90 days for the same or a similar chief complaint as the initial ED visit. Spontaneous revisits were defined as patient-initiated encounters, excluding those directed by health care providers.

#### Secondary Outcomes

Secondary outcomes included the rate of outpatient follow-up and the number of days to the first outpatient evaluation following ED discharge.

#### Data Collection

Patient data were collected through manual chart review using the Epic EMR. Variables collected include patient demographics (age, gender, race, and ethnicity), Emergency Severity Index (ESI) at the time of the ED visit, and 6 common comorbidities (coronary artery disease, chronic obstructive pulmonary disease, congestive heart failure, hypertension, diabetes mellitus, and transplant status). Additional data included ED visit details (date and care received), follow-up appointments (virtual transitional care visits, primary care, and specialist visits), and, if applicable, dates and chief complaints for ED revisits.

### Statistical Analysis

Descriptive statistics were calculated for baseline characteristics. A generalized linear model was used to analyze the continuous outcome (time to first outpatient evaluation in days), and a logistic regression model was built for the binary outcome (occurrence of spontaneous ED revisits within 90 days). Multivariable models were adjusted for patient demographics (age, gender, race, and ethnicity) and clinical characteristics (ESI and comorbidities). Statistical significance was established at *P*<.05. All analyses were performed in R (version 4.4.1; R Foundation for Statistical Computing).

### Ethical Considerations

This study was reviewed and classified as a quality improvement initiative and “Not Human Research” by the health system’s institutional review board, exempting it from full human subjects research approval under institutional guidelines for quality improvement activities. No informed consent was required as this study involved retrospective analysis of existing medical records and quality improvement data. Patients received standard clinical care and routine follow-up outreach as part of established care protocols. All patient data were deidentified for analysis purposes. Data collection was conducted through the institution’s secure Epic EMR system with appropriate access controls and institutional data security protocols in place to safeguard patient information. No compensation was provided to participants as this was a retrospective analysis of routine clinical care data without direct patient participation.

## Results

### Study Population and Engagement Cascade

Over the study period, the 4 EDs recorded 111,648 total encounters, including 38,155 admissions, 68,115 discharges, 741 transfers, and 4637 other dispositions. Of the 68,115 discharges, 51.76% (35,256/68,115) were assigned to the virtual transitional care pathway. This percentage reflects the program’s phased rollout, in which eligibility was initially restricted to selected freestanding ECCs and 4 diagnoses before expansion to the main academic ED and all diagnoses. Of the 35,256 pathway assignments, 82.54% (29,100/35,256) received an SMS text message. The remaining cases were excluded because patients either had no phone number on file or had a landline number ineligible for text delivery.

**Figure 2.**
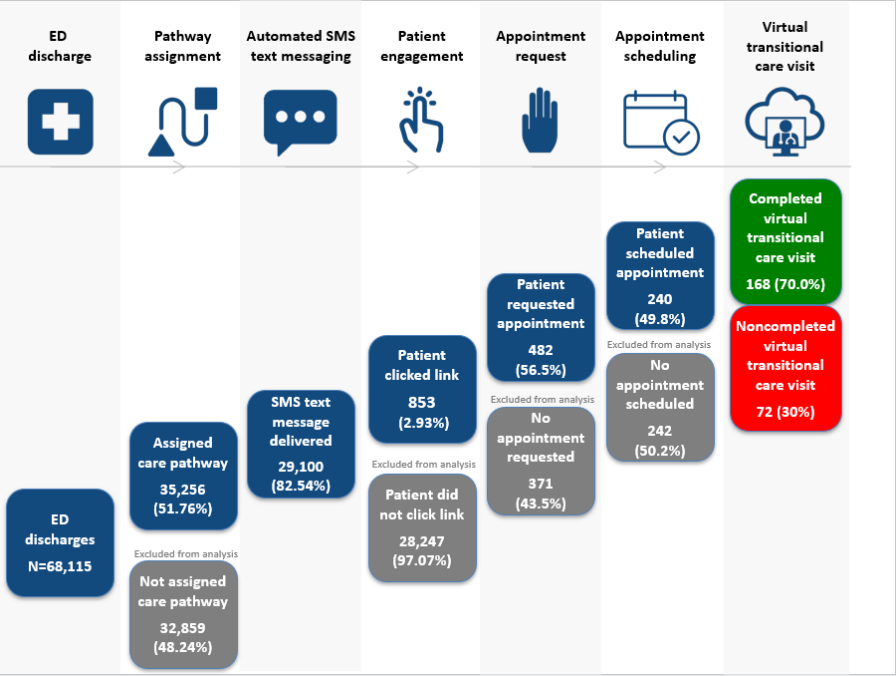
Emergency department (ED) discharge pathway and group classification by virtual transitional care visit completion status.

Among text recipients, 2.93% (853/29,100) clicked the scheduling link. Among those who clicked the link, 56.5% (482/853) requested a virtual transitional care visit, and 49.8% (240/482) of requesters successfully scheduled an appointment. Ultimately, 168 patients (70% of those scheduled) completed the visit, while 72 (30%) did not. The stepwise retention of patients through this engagement pathway is presented in Table 1. Figure 2 provides a visual summary of the engagement pathway and illustrates how patients were categorized into the completed and noncompleted virtual transitional care visit groups.

**Table 1.** Patient engagement cascade following emergency department (ED) discharge (N=68,115).

Event in engagement cascade	Total, n	Prior step, n/N (%)	Total ED discharges (%)
Total ED discharges	68,115	N/A^a^	100
Assigned to virtual transitional care pathway	35,256	51.76 (35,256/68,115)	51.76
Received automated text message	29,100	82.54 (29,100/35,256)	42.72
Clicked scheduling link	853	2.93 (853/29,100)	1.25
Requested a virtual transitional care visit	482	56.51 (482/853)	0.71
Scheduled an appointment	240	49.79 (240/482)	0.35
Completed (completed virtual transitional care visit group)	168	70.00 (168/240)	0.25
Did not complete visit (noncompleted virtual transitional care visit group)	72	30.00 (72/240)	0.11

a N/A: not applicable.

### Engagement Funnel Analysis

The virtual transitional care visit program demonstrated substantial attrition at each engagement step. Of the 68,115 total discharged patients, only 0.25% (168/68,115) ultimately completed a virtual transitional care visit. The largest drop-off occurred at the initial SMS text messaging stage, where 97.07% (28,247/29,100) of recipients did not click the scheduling link. Among those who did engage, conversion rates improved: 56.5% (482/853) of link-clickers requested visits, 49.8% (240/482) of requesters successfully scheduled appointments, and 70% (168/240) of scheduled patients completed their visits. This pattern indicates that while initial engagement was challenging, patients who demonstrated early interest showed progressively higher commitment to completing the care pathway.

### Baseline Characteristics

As shown in Table 2, patients in both completed and noncompleted groups were predominantly women (71.4% and 72.2%, respectively). The mean age was similar between groups (54.7, SD 17.7 y completed group versus 52.7, SD 21.6 y noncompleted group*; P*=.55). There were no significant differences between the groups in terms of race, ethnicity, or clinical comorbidities. The mean ESI was identical in both groups at 3.3 (SD 0.6), indicating comparable clinical acuity.

**Table 2.** Baseline demographic and clinical characteristics.

Characteristic	Completed (n=168)	Noncompleted (n=72)	*P* value
Age (y), mean (SD)	54.7 (17.7)	52.7 (21.6)	.55
Gender, n (%)			
Women	120 (71.4)	52 (72.2)	>.99
Men	48 (28.6)	20 (27.8)	>.99
Race, n (%)			.19
African American	55 (32.7)	33 (45.8)	
Asian	8 (4.8)	4 (5.6)	
Other	6 (3.6)	2 (2.8)	
White	99 (58.9)	33 (45.8)	
Ethnicity, n (%)			
Non-Hispanic	141 (84.9)	63 (88.7)	.54
Hispanic	25 (15.1)	8 (11.3)	.54
Comorbidities, n (%)			
CAD^a^	147 (87.5)	58 (80.6)	.17
CHF^b^	159 (94.6)	64 (88.9)	.17
COPD^c^	164 (97.6)	70 (97.2)	>.99
Hypertension	74 (44)	34 (47.2)	.67
DM^d^	132 (78.6)	58 (80.6)	.86
Transplant	166 (98.8)	72 (100)	>.99
Emergency Severity Index, mean (SD)	3.3 (0.6)	3.3 (0.6)	.49

a CAD: coronary artery disease.

b CHF: congestive heart failure.

c COPD: chronic obstructive pulmonary disease.

d DM: diabetes mellitus.

At the time of their ED visit, most patients in both groups had at least 1 provider of record. Among patients in the completed group, 26.2% (44/168) were linked to a primary care physician within the health system and 28% (47/168) to an outside primary care physician. In contrast, 23.6% (17/72) of the noncompleted group were linked to a primary care physician within the health system and 20.8% (15/72) to an outside primary care physician. From both groups, 13 (7.7%) patients in the completed group and 11 (15.2%) in the noncompleted group had no provider listed. Although not statistically analyzed, these differences may reflect broader patterns in health care access and engagement.

### Clinical Interventions Delivered During Virtual Transitional Care Visits

Out of 168 patients in the completed group, 106 (63.1%) received additional care from VUC nurse practitioners during their visit. The most common interventions included issuing a new prescription (39/113, 34.5%) and directing patients back to the ED (28/113, 24.8%). Other actions included medication adjustments (14/113, 12.4%), referrals to specialists (15/113, 13.3%) or primary care providers (9/113, 7.9%), and instructions to return to urgent care (4/113, 3.5%). A small number of patients received other support such as documentation or clarification of care instructions (3/113, 2.7%). Because some patients received more than one intervention, the total number of interventions (N=113) exceeds the number of patients who received them (106/168, 63.1%). The remaining 62 (36.9%) patients were evaluated but did not require any further clinical action; their concerns were either resolved, self-limited, or deemed appropriate for routine outpatient follow-up without nurse practitioner–initiated intervention. All patients, regardless of whether a clinical intervention was provided, were encouraged to follow up with their outpatient provider.

Notably, 24.8% (28/113) of patients who received a clinical intervention—16.7% (28/168) of all patients in the completed group—were directed back to the ED by nurse practitioners, representing a substantial proportion that required escalation to higher-level care. Of the 28 (16.7%) patients directed to return to the ED, 18 (64%) followed through with this recommendation, indicating good patient adherence to clinical guidance provided during virtual visits.

### Outcomes

We assessed three outcomes: spontaneous ED revisits within 90 days of discharge, outpatient follow-up rates, and time to first outpatient evaluation.

Patients (29%, 21/72) in the noncompleted group were almost twice as likely to revisit the ED within 90 days compared to the completed group (18.7%, 28/150). This difference remained significant in a multivariate logistic regression model adjusting for demographics, comorbidities, and ESI. As shown in Table 3, patients who did not complete a virtual transitional care visit were more than twice as likely to return to the ED within 90 days (odds ratio 2.11; 95% CI 1.02‐4.33; *P*=.04). Among the completed group patients who were advised by a nurse practitioner to return to the ED, 18 out of 28 (64%) followed through.

**Table 3.** Multivariate logistic regression: predictors of spontaneous emergency department (ED) revisits within 90 days.

Variables	Odds ratio (95% CI)	*P* value
(Intercept)	0.31 (0.08-1.22)	.09
Group		
Noncompleted (ref=completed)	2.11 (1.02-4.33)	.04
Age (y)	0.99 (0.97-1.02)	.48
Gender		
Men (ref=women)	0.75 (0.34-1.7)	.50
Race		
Asian (ref=White)	0 (0-inf)^a^	.99
Black (ref=White)	0.58 (0.26-1.32)	.19
Others (ref=White)	1.09 (0.11-11.08)	.94
Ethnicity		
Hispanics (ref=non-Hispanics)	0.67 (0.21-2.12)	.49
Comorbidities (yes)		
Congestive heart failure	0.68 (0.15-3.09)	.62
Coronary artery disease	0.97 (0.36-2.63)	.95
Chronic obstructive pulmonary disease	3.43 (0.56-20.9)	.18
Diabetes mellitus	1.32 (0.53-3.29)	.55
Hypertension	1.85 (0.75-4.54)	.18
Transplant	4.94 (0.24-100.17)	.30

a There is no difference between White versus Asian patients with the outcome (ED visit), that is, they are extremely similar or have extreme similar patterns.

On average, patients in the completed group completed their virtual transitional care visit within 3.83 (SD 1.22) days. They were also twice as likely to complete outpatient follow-up after ED visit compared to the noncompleted group (48.6% vs 30%, respectively; odds ratio 2.17; 95% CI 1.23‐3.83; *P*=.01).

Although patients in the completed group were more likely to follow up, there were no significant differences in the timing of the first outpatient evaluation between the groups. As shown in Table 4**,** the mean time to first outpatient visit was 15.7 (SD 19.0) days in the noncompleted group versus 19.8 (SD 20.7) days in the completed group (Δ*β*=−1.93, 95% CI −10.09 to 6.42; *P*=.65).

**Table 4.** Multivariate linear regression: timing of first outpatient evaluation.

Variables	Estimated days (95% CI)	*P* value
(Intercept)	9.69 (−6.68 to 26.05)	.25
Group		
Noncompleted (ref=completed)	−1.93 (−10.29 to 6.42)	.65
Age (y)	0.23 (−0.03 to 0.49)	.09
Gender		
Men (ref=women)	2.80 (−5.74 to 11.35)	.52
Race		
Asian (ref=White)	2.97 (−14.60 to 20.54)	.74
Black (ref=White)	−2.66 (−11.27 to 5.94)	.55
Others (ref=White)	12.22 (−9.99 to 34.43)	.28
Ethnicity		
Hispanics (ref=non-Hispanics)	−3.17 (−15.92 to 9.58)	.63
Comorbidities (yes)		
Congestive heart failure	−11.18 (−25.34 to 2.98)	.12
Coronary artery disease	−5.17 (−14.71 to 4.38)	.29
Chronic obstructive pulmonary disease	5.00 (−14.39 to 24.39)	.61
Diabetes mellitus	0.12 (−9.20 to 9.44)	.98
Hypertension	−3.84 (−13.00 to 5.32)	.41
Transplant	2.99 (−28.07 to 34.04)	.85

## Discussion

### Principal Findings

Among 68,115 ED-discharged patients, we implemented a TCI using automated SMS text messaging linked to a virtual transitional care program. While only 0.25% (168/68,115) of discharged patients ultimately completed a virtual transitional care visit, those who did demonstrated significantly improved outcomes: 53% lower odds of 90-day ED revisits and doubled rates of outpatient follow-up completion compared to patients who scheduled but did not attend virtual transitional care visits. These findings suggest the intervention effectively identified and served patients with ongoing postdischarge needs while maintaining high operational efficiency through patient self-selection.

To our knowledge, this study is the first to describe a virtual TCI that integrates automated text messaging with virtual transitional care visits, representing a novel application of existing virtual care infrastructure for postdischarge care coordination.

### Impact on ED Revisits

Our finding was a lower rate of spontaneous ED revisits among patients who completed a virtual transitional care visit compared to the noncompleted group. By excluding provider-directed revisits, we isolated the effect of postdischarge care through virtual transitional care visits. These results contrast with findings from Bressman et al [14], whose similar SMS text messaging TCI did not reduce ED revisit rates within 30 days. One probable factor for this variance is that their population was limited to patients who had already established care at primary care practices within their health system. We build on their work by expanding our TCI’s target population to all patients discharged from the ED, including those without a primary care provider and those not established in our health system. This allowed our program to reach a broader, more diverse patient population with varying health care access, which may explain the differential impact on revisit rates.

The substantial proportion of patients (28/168, 16.7% of the completed group) who were directed back to the ED during virtual transitional care visits deserves particular attention. This rate is notably higher than typical urgent care referral patterns and likely reflects the postdischarge context, in which patients may have evolving or incompletely resolved conditions. Rather than representing a program failure, this pattern suggests successful identification of patients requiring escalated care who might otherwise have delayed seeking appropriate treatment. 18 out of 28 (64%) patients adhered to instructions to return to the ED, underscoring the clinical value of structured postdischarge follow-up. The case examples in Multimedia Appendix 1 illustrate how virtual reassessment enabled timely recognition of clinical deterioration, potentially preventing more serious complications. However, this high referral rate also highlights the complexity of post-ED discharge care and implies that a significant subset of discharged patients may benefit from more intensive monitoring or alternative care models.

### Outpatient Follow-Up Engagement

Our major finding was that completed participants were significantly more likely to complete an outpatient follow-up visit compared to those in the noncompleted group. Similar trends have been reported by Bhandari et al [15] and Costantino et al [6]. However, this finding should be interpreted cautiously given potential selection bias, as patients who completed virtual transitional care visits likely represent a more engaged population.

### Engagement Patterns and Gender Distribution

While 72.0% (121/168) of the patients who used virtual transitional care visits were women, a distribution consistent with higher telehealth uptake among women during the VUC program pilot study [13] and prior studies [16-18], this pattern is especially notable given the ED context. It suggests that gender-based consumer preferences for timely, convenient care persist even in postacute scenarios, potentially shaping who engages with digital transitional care models.

Despite this observed preference pattern, overall engagement with the virtual transitional care program was lower than expected. Only 2.93% (853/29,100) of SMS text messaging recipients clicked the virtual transitional care link, and just over half of those (482/853, 56.5%) requested a visit. This low engagement rate contrasts with the higher engagement observed in prior studies using bidirectional texting, which allowed patients to respond to the SMS text messaging outreach [10,14]. One possible contributor to the low uptake was the wording of the SMS text messaging: patients without “remaining concerns or issues” may have been unlikely to click the scheduling link, and they did not have the opportunity to ask clarifying questions as would have been possible in a bidirectional model. While the automated unidirectional texting approach helped minimize staffing burden and operational complexity, it limited real-time responsiveness. Future iterations of the program may benefit from a more seamless, patient-driven experience, such as direct scheduling or on-demand access, as well as exploration of bidirectional texting models.

### Workflow Considerations and Operational Efficiency

Additional patients were lost when PSRs reached out via phone to confirm the appointment request and schedule a virtual transitional care visit. This outcome was somewhat anticipated, as telephone outreach necessitates the patient’s availability at the time the PSR places the call. Based on operational workflows, patients typically waited 1 to 2 days between clicking the link and completing their scheduled virtual transitional care visit. This added step and subsequent delays may have introduced friction or led to disengagement, particularly for those with lower-acuity concerns or limited availability.

However, this step was included in the program workflow to address concerns about potentially unmanageable, high ED-discharge patient volume flowing into the virtual transitional care program (68,115 discharges during the study period). By having PSRs confirm patients’ remaining concerns and schedule virtual transitional care visits, it was also possible to distribute the patient volume throughout each day, making patient visits more manageable for VUC clinicians. PSRs also supported care navigation by redirecting patients with concerns outside of the scope of virtual transitional care, such as requests for controlled substances, to more appropriate care settings. Once scheduled, 70% (168/240) of patients successfully completed their virtual transitional care visit. The high adherence rate indicates this multistep process effectively triages patients with ongoing needs and routes them to appropriate care.

### Program Reach and Resource Efficiency

The engagement challenges revealed by the cascade analysis demonstrate both limitations and strengths of this approach. With only 1 in 400 discharged patients ultimately completing a virtual transitional care visit, the program’s reach was more limited than anticipated. The 97% nonresponse rate to text messages represents the most significant barrier and likely reflects multiple factors: SMS text messaging fatigue in health care settings, competing priorities during postdischarge recovery, resolution of concerns before follow-up, and potential technological or literacy barriers.

From an operational perspective, the low engagement rate meant that the 29,100 (82.54%) SMS text messages sent ultimately generated only 168 completed visits (0.58% conversion), making this intervention highly efficient in terms of provider time and resources. Once patients demonstrated initial engagement by clicking the link, conversion rates improved substantially. This suggests the program successfully identified and served patients with the highest need for postdischarge support while avoiding overwhelming virtual transitional care capacity.

### Outpatient Care Timing

While the program appears to improve the likelihood of follow-up, there was no significant difference in the timing of the first outpatient evaluation after the ED visit between completed and noncompleted groups. This finding may reflect the retrospective nature of our study, which prevented differentiation between appointments scheduled before versus after the ED visit, and our inclusion of all specialty providers rather than focusing on visits related to the presenting complaint.

### Limitations

This study has several important limitations that should be considered when interpreting the findings.

#### Study Design and Comparison Groups

First, we did not compare the completed group to all ED-discharged patients but instead focused on patients who had both requested and scheduled a virtual transitional care visit. While this cohort design allowed for a more targeted comparison of clinical outcomes, as both groups expressed lingering concerns after their ED discharge, it limits the generalizability of our findings to the broader ED discharge population. The overall sample size (240 patients) also reduced statistical power to detect differences between groups.

The small sample sizes within racial and ethnic subgroups further limited our ability to detect meaningful differences between the completed and noncompleted groups. While we observed variations in racial distribution (White: 99/168, 58.9% vs 33/72, 45.8%; African American: 55/168, 32.7% vs 33/72, 45.8%), the restricted sample size constrained statistical power for subgroup analyses. This represents a missed opportunity for robust statistical inference regarding potential disparities in program access, engagement, or outcomes. Future studies with larger samples are needed to adequately assess whether racial or ethnic factors influence engagement with virtual transitional care visits.

Another important limitation is our inability to compare outcomes between the completed group and the larger population of patients who received automated SMS text messaging but did not access the scheduling link (28,247 patients, representing 97.07% of text recipients). Even the noncompleted group demonstrated engagement by clicking the link and requesting virtual transitional care visits, representing a higher level of activation than the nonclicking population. We did not collect or analyze demographic or clinical characteristics of patients who received texts but did not engage with the scheduling link, which could have provided valuable insights into differences between engaged and nonengaged populations. Furthermore, we did not conduct qualitative interviews to explore reasons for nonengagement. These limitations restrict our understanding of the full spectrum of patient engagement and reduce our ability to evaluate the program’s impact across all text recipients.

#### Selection Bias and Patient Engagement

Second, our findings are significantly influenced by selection bias. While both the completed and noncompleted groups demonstrated initial motivation by requesting and scheduling virtual transitional care visits, patients who actually completed visits may still represent a more activated or health-engaged subgroup compared to those who scheduled but did not attend. This could partially explain the higher observed rate of outpatient follow-up among completed patients. However, the more significant limitation is our inability to compare either engaged group to the 28,247 (97.07%) text recipients who never clicked the scheduling link, representing the vast majority of the ED discharge population.

The low overall engagement with the intervention (only 853/29,100, 2.93% of text recipients clicked the link) raises important questions about program reach. We did not track SMS text messaging read receipts, so it remains unclear whether the 97% nonresponse rate reflects unread messages, conscious decisions not to engage, or technological barriers. This limitation prevents us from distinguishing between patients who were unaware of the opportunity versus those who actively chose not to participate.

It is possible that some patients failed to complete their scheduled virtual transitional care visit because they were already experiencing worsening symptoms or barriers to care, factors that may have directly contributed to their subsequent ED return. As such, the association between visit completion and improved outcomes should be interpreted with caution, given potential confounding by severity, access, or health literacy.

#### Data Collection and Measurement Limitations

Third, our study’s retrospective design introduced limitations in data collection and measurement. It was not possible to differentiate through chart review whether the outpatient appointment was scheduled before or after the ED visit. In addition, we included providers across all specialties for the first outpatient appointment, making it unclear whether patients were seen for the same chief complaint that brought them to the ED or for unrelated issues. For example, if a patient presented to the ED with chest pain and saw an otolaryngologist 3 days later, this would still be recorded as an outpatient follow-up at 3 days after discharge. This limitation may have contributed to the lack of observed difference in follow-up timing between the 2 groups.

External acute care visits, outpatient visits, and other postdischarge outreach could have been missed if they were not available through Epic Care Everywhere for retrospective chart review; however, this would have occurred randomly in both groups and is unlikely to have systematically biased our results.

We did not systematically document PSR contact rates during phone outreach, though anecdotal reports suggest approximately 50% of patients who clicked the scheduling link did not answer subsequent phone calls. This represents an unmeasured but potentially significant source of attrition that limits our understanding of barriers to care access among initially engaged patients and may underestimate true demand for virtual transitional care services.

#### Accessibility and Generalizability Concerns

This TCI design may have also excluded patients facing language and technological barriers. Those who cannot read English would have been unable to understand the SMS text messaging and would have required additional assistance to participate. Patients with technological difficulties, such as unreliable internet access or unfamiliarity with telehealth, may have been less inclined to click on the link. Alternatively, it is possible that patients who did not click on the virtual transitional visit link had no remaining issues after their ED discharge. In addition, this study was conducted within a single health system, which may limit the generalizability of our findings to other health care settings with different patient populations, technological infrastructure, or staffing models. While this TCI framework demonstrates potential for scalability across diverse health care settings, successful implementation would require customization to accommodate local patient populations, technological capabilities, language preferences, and existing care delivery models. Health systems considering adoption should adapt messaging content, scheduling workflows, and clinical protocols to align with their specific patient demographics and operational constraints.

### Future Work

Building on the promising findings of this study, efforts are underway to expand the virtual transitional care program across all hospitals and affiliated freestanding ECCs within our health system. Prior to broader implementation, optimizing program workflows should be considered. The current multistep process—requiring patients to wait for a PSR to call and schedule a visit after they engage with the SMS text messaging—may have introduced friction and contributed to lower overall participation. While most virtual transitional care visits were scheduled for the day after patient outreach, this 1- to 2-day delay may not align with the expectations of patients seeking more immediate care access. Streamlining this process through integrated self-scheduling or offering on-demand virtual transitional care visits could reduce attrition. In addition, incorporating a bidirectional texting model may enhance responsiveness, foster more dynamic communication, and increase overall engagement, an approach that has shown promise in prior postdischarge care studies. Integrating patient language preferences from Epic into the messaging workflow and tailoring communication accordingly could further extend the program’s reach to linguistically diverse populations.

#### Prior ED Use Patterns

An important area for future investigation involves examining baseline ED use patterns among patients who complete virtual transitional care visits versus those who do not. Understanding whether patients who engage with postdischarge virtual care have different historical patterns of acute care use could contribute key knowledge about patient activation, health-seeking behaviors, and care preferences. Such analysis could help identify whether the program primarily attracts patients who are already engaged with the health care system or successfully reaches patients with traditionally poor care continuity. This information would be crucial for targeting interventions and understanding the mechanisms by which virtual transitional care visits influence subsequent health care use.

#### Qualitative Research Opportunities

Future studies should incorporate qualitative methods to better understand patient motivations for engagement or nonengagement with postdischarge SMS text messaging and virtual transitional care programs. Interviews with patients who do not click scheduling links could reveal whether nonresponse reflects resolved concerns, technological barriers, competing priorities, or other factors. Similarly, qualitative work with patients who schedule but do not complete virtual transitional care visits could identify modifiable barriers to care completion. This information would be invaluable for refining program design and improving patient engagement strategies.

#### Validation and Long-Term Impact Studies

While this study offers promising early insights, further research is needed to validate the impact of the virtual transitional care program on acute care reuse using a larger sample size. Advanced analytical techniques such as latent class analysis may help identify patient subgroups with differential responses to virtual TCIs, enabling more targeted and effective program design. The long-term impact of this virtual TCI remains uncertain, particularly with regard to hospital readmission rates, continuity of care from new referrals, and patient satisfaction. If the program demonstrates positive outcomes in these areas, it could offer valuable administrative insights by potentially reducing readmission-related financial penalties and improving patient retention within the health system. Notably, the program was implemented without additional resources or staffing, indicating that meaningful patient engagement and clinical follow-up can be achieved using existing infrastructure. This is a key consideration for long-term sustainability and scalability. Addressing these factors will help refine virtual TCI models and guide broader implementation strategies in postacute transitional care.

### Conclusions

The virtual TCI, combining automated SMS text messaging with virtual follow-up visits, demonstrated significant clinical benefits among engaged patients: those who scheduled and completed virtual transitional care visits were less likely to return to the ED within 90 days compared to those who scheduled but did not complete visits. However, given that only 2.9% (853/29,100) of eligible patients engaged with the virtual TCI initially, the broader population-level impact remains limited. While the program demonstrated clinical benefits among engaged patients, the substantial engagement challenges highlight the need for both optimizing the virtual TCI delivery for the small subset who do engage and also developing approaches to expand engagement more broadly across the ED discharge population. In addition, the program’s multistep scheduling workflow, requiring phone-based appointment confirmation, may have introduced friction that limited overall participation. Future work should prioritize both streamlined virtual transitional care processes for engaged patients and innovative outreach strategies to reach the 97% who did not initially respond to the SMS text messaging component of the intervention. Streamlined, patient-driven scheduling and bidirectional communication models may further enhance engagement and scalability. Further research is needed to validate the program’s impact across larger and more diverse populations.

## Supplementary material

10.2196/77973 Multimedia Appendix 1 Clinical case examples: emergency department (ED) return and admission following virtual transitional care visit.

10.2196/77973 Multimedia Appendix 2 Virtual transitional care program deidentified dataset for statistical analysis.

## Abbreviations

ECC: emergency care center
ED: emergency department
EMR: electronic medical record
ESI: Emergency Severity Index
PSR: patient service representative
TCI: transitional care intervention
VUC: virtual urgent care
