# Supplementary material for: Implementation and Evaluation of a Virtual Transitional Care Intervention Using Automated Text Messaging and Virtual Visits After Emergency Department Discharges: Retrospective Cohort Study
Source: JMIR Mhealth Uhealth. 2025 Oct 29;13:e77973. doi: 10.2196/77973 (PMC12571198; doi:10.2196/77973)
Supplement: Multimedia Appendix 1 [file mhealth-v13-e77973-s001.docx]

**Appendix 1.** *Clinical Case Examples: ED Return and Admission Following Virtual Transitional Care Visit*

Out of 19 patients who were directed to return to the ED as directed by virtual urgent care (VUC) practitioners (NPs), 11 (57.9%) were admitted to the hospital. Below are three illustrative cases in which patients who experienced clinical deterioration or worsening symptoms that were identified by the virtual care follow-up visit and required inpatient admission.

**Case 1**: A 42-year-old female with a history of Hashimoto’s thyroiditis and hypercholesterolemia initially presented to the ED with three days of subjective fever, chills, nausea, vomiting, diarrhea, and myalgia following travel to San Antonio. Labs were ordered to rule out acute infectious pathology. The patient was diagnosed with presumed gastroenteritis and discharged with outpatient follow-up instructions. Four days later, during her scheduled virtual transitional care visit, she reported worsening malaise and new-onset fever. She was referred back to the ED, where she was found to have severe sepsis. Labs revealed elevated Rickettsia typhus serologies and imaging showed multifocal pneumonia. She was admitted, treated with targeted antibiotics, and later discharged in stable condition.

**Case 2:** A 41-year-old male with end-stage renal disease on peritoneal dialysis (PD), type 2 diabetes mellitus, hypertension, diastolic heart failure, and HIV presented to the ED with left thigh pain that had caused him to miss dialysis sessions. The patient had previously visited an outside ED, where he underwent an ultrasound of his left lower extremity (LLE) to rule out acute venous thromboembolism. The result was reportedly negative, and a repeat ultrasound of the left lower extremity (LLE) ruled out acute venous thromboembolism, and labs showed no emergent dialysis need. He was discharged. Three days later, during the virtual transitional care visit, he reported worsening left thigh pain and a new mass. He was referred back to the ED where he was admitted for further workup. MRI revealed multifocal myositis of the adductor muscles and distal myonecrosis of the adductor magnus, likely secondary to uncontrolled diabetes. He was treated and discharged in stable condition.

**Case 3:** An 85-year-old female with a history of Alzheimer’s dementia, recurrent urinary tract infections (UTIs), and atrial fibrillation (status post-cardiac pacemaker placement) initially presented to the ED with weakness and chills while on cefuroxime for a UTI. She was accompanied by her sister. The patient denied having a fever at home, but the patient’s sister reported that she had experienced episodes of diarrhea prior to the UTI but that these had resolved. The patient was found to be febrile in the ED, and the care team recommended observation, but the patient wished to go home after her fever resolved and her other symptoms improved. She was discharged with instructions to follow up with her primary care physician. Three days later, during her virtual urgent care follow-up appointment, the patient reported new-onset bloody diarrhea. She was referred back to the ED and was found to have acute blood loss anemia, with her hemoglobin dropping from 11.9 g/dL to 8.4 g/dL over three days. She was admitted and later discharged in stable condition.
